# Effects of Cold Rolling or Precipitation Hardening Treatment on the Microstructure, Mechanical Properties, and Corrosion Resistance of Ti-Rich Metastable Medium-Entropy Alloys

**DOI:** 10.3390/ma16247561

**Published:** 2023-12-08

**Authors:** Hsueh-Chuan Hsu, Ka-Kin Wong, Shih-Ching Wu, Chun-Yu Huang, Wen-Fu Ho

**Affiliations:** 1 Department of Dental Technology and Materials Science, Central Taiwan University of Science and Technology, Taichung 40601, Taiwan; hchsu@ctust.edu.tw (H.-C.H.); scwu@ctust.edu.tw (S.-C.W.); 2 Department of Chemical and Materials Engineering, National University of Kaohsiung, Kaohsiung 81148, Taiwan; m1075616@mail.nuk.edu.tw (K.-K.W.); wkk2020@nuk.edu.tw (C.-Y.H.)

**Keywords:** medium-entropy alloys, metastable, cold rolling, precipitation hardening, mechanical properties, corrosion behavior

## Abstract

Titanium-rich metastable medium-entropy alloys, designed for low elastic moduli, sacrifice strength. However, enhancing their mechanical strength is crucial for bio-implant applications. This study aims to enhance the mechanical properties and corrosion resistance of a metastable Ti_80_–Nb_10_–Mo_5_–Sn_5_ medium-entropy alloy using various treatments, including cold rolling (at 50% and 75% reduction) and precipitation hardening (at room temperature, 150 °C, 350 °C, 550 °C, and 750 °C). The results showed that the alloy underwent a stress-induced martensitic transformation during the rolling process. Notably, the α phase was precipitated in the β grain boundaries after 30 days of precipitation hardening at room temperature. The yield strengths of the alloy increased by 51% and 281.9% after room-temperature precipitation and 75% cold rolling, respectively. In potentiodynamic corrosion tests conducted in phosphate-buffered saline solution, the pitting potentials of the alloy treated using various conditions were higher than 1.8 V, and no pitting holes were observed on the surface of the alloys. The surface oxide layer of the alloy was primarily composed of TiO_2_, Nb_2_O_5_, MoO_3_, and SnO_2_, contributing to the alloy’s exceptional corrosion and pitting resistance. The 75% rolled Ti_80_–Nb_10_–Mo_5_–Sn_5_ demonstrates exceptional mechanical properties and high corrosion resistance, positioning it as a promising bio-implant candidate.

## 1. Introduction

In recent years, Ti-rich metastable β high-entropy alloys (HEAs) and medium-entropy alloys (MEAs) have garnered significant attention in the field of biomedical materials [1,2,3,4,5,6,7,8,9]. These alloys are known for their high mechanical strength and low elastic moduli. A key research goal is to improve the strengths of MEAs while maintaining their low elastic moduli. Severe plastic deformation can achieve grain refinement in conventional Ti alloys, which not only greatly increases their strength but also retains their original low elastic modulus [10]. Many studies have adopted cold rolling to produce grain refinement to improve the mechanical properties of HEAs/MEAs, such as Hf–Nb–Ta–Ti–Zr [11], Al_0.25_–Co–Cr–Fe–Ni [12], Cr–Co–Ni [13], Al_4_–Nb_8_–Ti_50_–Mo_4_–Zr_34_ [14], Ti_65_–(Al–Cr–Nb)_35_ [15], and Zr_35_–Ti_30_–Nb_20_–Al_10_–Ta_5_ [16]. The presence of an ultrafine grain structure not only provides the alloy with a high mechanical strength but also elevates its corrosion resistance [17,18]. Furthermore, when a metastable β HEA/MEA is subjected to external stress, a stress-induced martensitic (SIM) transformation may occur, resulting in a partial transformation of the β phase into the α″ phase [19,20]. After SIM transformation, a large number of dislocations and nano-sized α″ precipitates are formed, resulting in a notable enhancement in the alloy’s strength [21]. However, an excessive amount of the α″ phase in an alloy can lead to a decrease in strength [22,23].

Precipitation hardening is a common method to enhance the mechanical properties of Ti alloys, HEAs, and MEAs [24,25,26]. Via control of the precipitation treatment, it is possible to regulate the size and distribution of the precipitated phases, allowing for adjustments in various alloy properties. Precipitation hardening treatment can be divided into single-step (direct heat treatment) and two-step (solution treatment + heat treatment) processes. Two-step precipitation hardening treatment can produce a finer and more uniformly distributed α phase in β-Ti alloys than the single-step process, which can significantly increase the yield strength, ductility, and corrosion resistance of Ti alloys [24]. Moreover, the fine and uniformly distributed α phase does not significantly increase the elastic moduli of Ti alloys [27]. Furthermore, the precipitated phases can fill surface defects, improving the corrosion resistance of Ti alloys [28,29]. A significant amount of research has been conducted on the precipitation hardening of high-entropy alloys [25,26,30,31,32]. However, limited literature exists concerning the precipitation hardening of biomedical HEAs or MEAs, particularly in terms of its impact on alloys’ structure, mechanical properties, and corrosion resistance.

The innovative aspect of this study lies in the comprehensive investigation into the impact of various processing methods, specifically cold rolling and heat treatment, on Ti_80_–Nb_10_–Mo_5_–Sn_5_. This study delves into the intricate relationship between its microstructure, mechanical strength, and corrosion resistance. By exploring the alterations in the microstructure caused by these processing methods, this study provides novel insights into optimizing the alloy’s performance. Additionally, the study aims to identify the most effective processing conditions to enhance both the mechanical strength and corrosion resistance of Ti_80_–Nb_10_–Mo_5_–Sn_5_, thereby advancing its potential applications in biomedical materials.

## 2. Materials and Methods

The metastable β structure of the Ti_80_–Nb_10_–Mo_5_–Sn_5_ (at%) MEA is achieved by adjusting the molybdenum equivalent ([Mo]_eq_) to 15 and the valence electron concentration (VEC) to 4.2, which results in the low elastic modulus of the alloy. The thermodynamic parameters detailed in Table 1 were determined using calculations based on the formulas referenced in [9]. These ingots were prepared using a commercial arc-melting vacuum pressure casting system (A-028, DAWNSHINE, Taichung, Taiwan) with pure Ti (99.7 wt%), Nb (99.95 wt%), Mo (99.95 wt%), and Sn (99.9 wt%). All ingots underwent re-melting and were flipped at least six times to ensure the homogeneity of the constituent elements. The initial dimensions of the Ti_80_–Nb_10_–Mo_5_–Sn_5_ ingots were 15 mm × 15 mm × 15 mm, and after casting, the samples measured 40 mm × 5 mm × 5 mm. The solution treatment of each sample was conducted in a tubular furnace (MTF 12/38/250, Carbolite Gero, Hope Valley, UK) at 900 °C for 15 min in an argon atmosphere, followed by rapid cooling in ice water. Subsequently, Ti_80_–Nb_10_–Mo_5_–Sn_5_ samples underwent cold rolling using a rolling machine (5TSB, Jong Yih Co., Kaohsiung, Taiwan) with a roll diameter of 15 cm. Reductions of 50% and 75% in the sample thickness were achieved using incremental passes of cold rolling, with each pass incrementally reducing the thickness by approximately 10%. Seven passes were required to achieve the 50% reduction, while a 75% reduction demanded thirteen passes of cold rolling. The precipitation hardening treatments were performed using a tubular furnace under an argon atmosphere at temperatures of 150 °C, 350 °C, 550 °C, or 700 °C for 15 min each. The natural precipitation hardening treatment was maintained at 25 °C for 30 days. The codes for the Ti_80_–Nb_10_–Mo_5_–Sn_5_ alloy under various conditions are summarized in Table 2.

X-ray diffraction (XRD) employing Cu-Kα radiation was carried out by using an XRD system (D8 Advance, Bruker, Karlsruhe, Germany) to perform phase analysis at 40 kV and 40 mA, with a scanning range of 2θ = 30–80°, a scanning speed of 4°/min, and a step size of 0.02°/step. Electron backscatter diffraction (EBSD) was used to analyze the grain orientation and phase structure (SU5000, Hitachi, Tokyo, Japan). In order to evaluate the mechanical properties of the samples under the flexural stresses that occur during human body movement, three-point bending tests were performed using a desktop mechanical tester (HT-2102AP, Hung Ta Instrument, Taichung, Taiwan). The procedures of the three-point bending experiments were in accordance with those described in previous works [33]. The yield strength was determined using a 0.2% strain offset from the stress–strain curve obtained during the bending test.

To evaluate the corrosion behavior of the Ti_80_–Nb_10_–Mo_5_–Sn_5_ sample, potentiodynamic polarization tests were conducted using a Potentiostat (PGSTAT12, Autolab, Utrecht, The Netherlands) in phosphate-buffered saline (PBS) at 37 °C and a pH of 7.4. The working electrode, reference electrode, and auxiliary electrode used were the Ti_80_–Nb_10_–Mo_5_–Sn_5_ sample, a silver chloride electrode (Ag/AgCl), and a platinum plate, respectively. Each specimen was tested using three individual samples to investigate their corrosion resistance. The exposed surface area of the working electrode was 10 mm × 10 mm × 1 mm. To ensure accuracy, the electrolyte solution was purged with nitrogen gas for 30 min prior to testing. The potentiodynamic polarization tests were initiated after the open circuit potential (OCP) had stabilized for an hour in the PBS solution. The scan rate and scan ranges were set at 0.001 V/s and −0.3 to 1.8 V, respectively. The PBS solution contained NaCl (8.0 g/L), KCl (0.2 g/L), Na_2_HPO_4_ (1.44 g/L), and KH_2_PO_4_ (0.24 g/L) [34]. The corrosion potentials and corrosion current densities were calculated using the Tafel extrapolation method. Following the potentiodynamic polarization tests, scanning electron microscopy (SEM; 6330, JEOL, Tokyo, Japan) was used to examine the microstructure of the alloys. Additionally, electrochemical impedance spectroscopy (EIS) measurements were conducted using an applied alternating current signal with a voltage amplitude of 10 mV in a frequency range from 10^5^ to 10^−2^ Hz, with measurements taken at the OCP after immersion in the electrolyte for 1 h. The EIS Spectrum Analyzer software was utilized to fit and analyze the experimental data. Moreover, X-ray photoelectron spectroscopy (XPS; JAMP-9500F, JEOL, Tokyo, Japan) was utilized to analyze the surface chemical compositions of the alloy after the potentiodynamic polarization tests.

## 3. Results and Discussion

### 3.1. Microstructure

**Figure 1:**
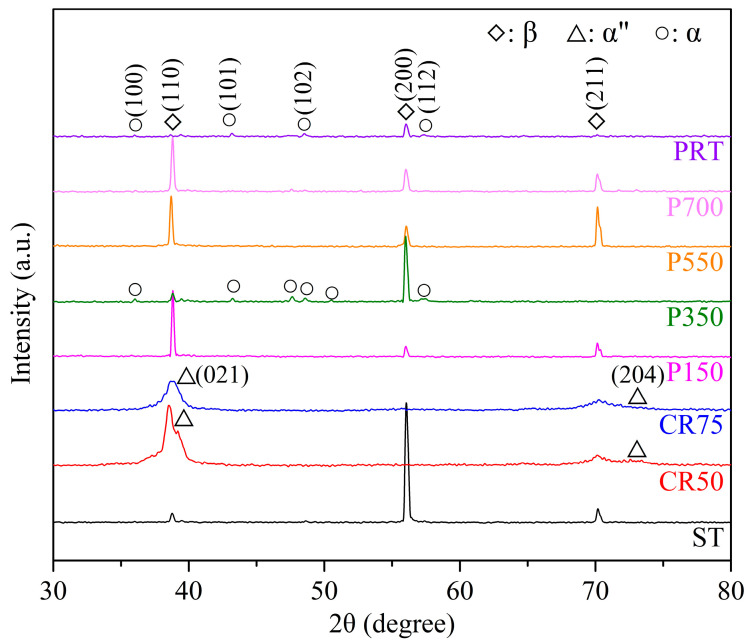
XRD patterns of Ti_80_–Nb_10_–Mo_5_–Sn_5_ alloy at various conditions.

The XRD patterns of Ti_80_–Nb_10_–Mo_5_–Sn_5_ under different conditions are presented in Figure 1. In the ST state, Ti_80_–Nb_10_–Mo_5_–Sn_5_ exhibited a single β phase, while after 50% and 75% cold rolling, a β + α″ phase was observed due to the SIM transformation. After the precipitation hardening treatment, the P150, P550, and P700 samples displayed a single β phase, while P350 and PRT showed a β + α phase. The alloy still presented a single β phase after low-temperature (150 °C) heat treatment, attributed to an insufficient heating time for α phase precipitation. Similarly, the high-temperature heat treatment (>550 °C) resulted in a single β phase due to the heating temperatures exceeding the β-phase transus temperature. In contrast, at room temperature, the precipitation hardening behavior of the Ti_80_–Nb_10_–Mo_5_–Sn_5_ was primarily attributed to the abundant vacancies accelerating atomic diffusion and α phase nucleation and growth [35]. The rapid cooling process during casting caused vacancies to remain in the alloy, while high-temperature solution treatment increased the equilibrium concentration of the vacancies [36].

**Figure 2:**
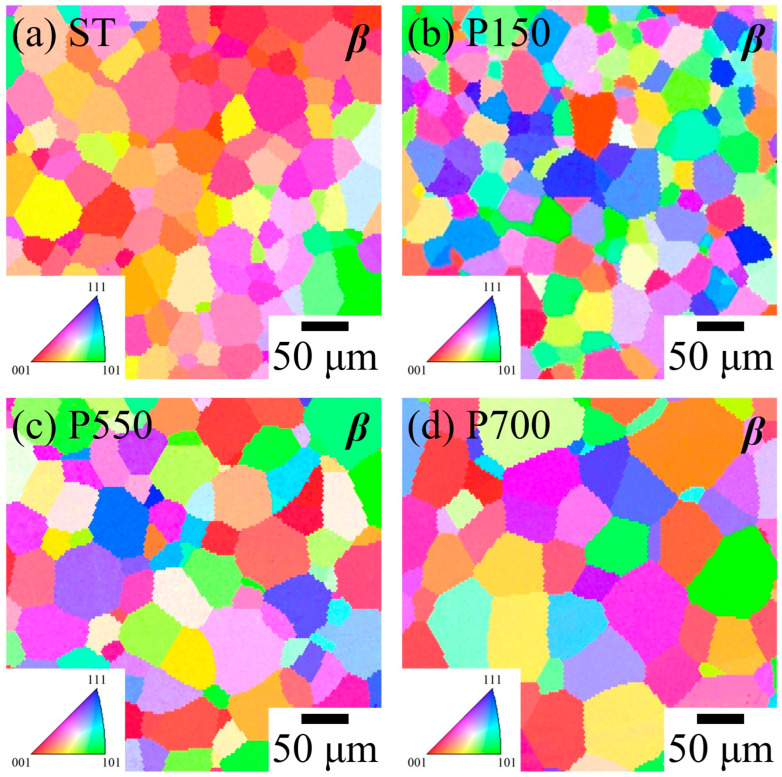
IPF-EBSD images of Ti_80_–Nb_10_–Mo_5_–Sn_5_ at various conditions with single β phase.

**Figure 3:**
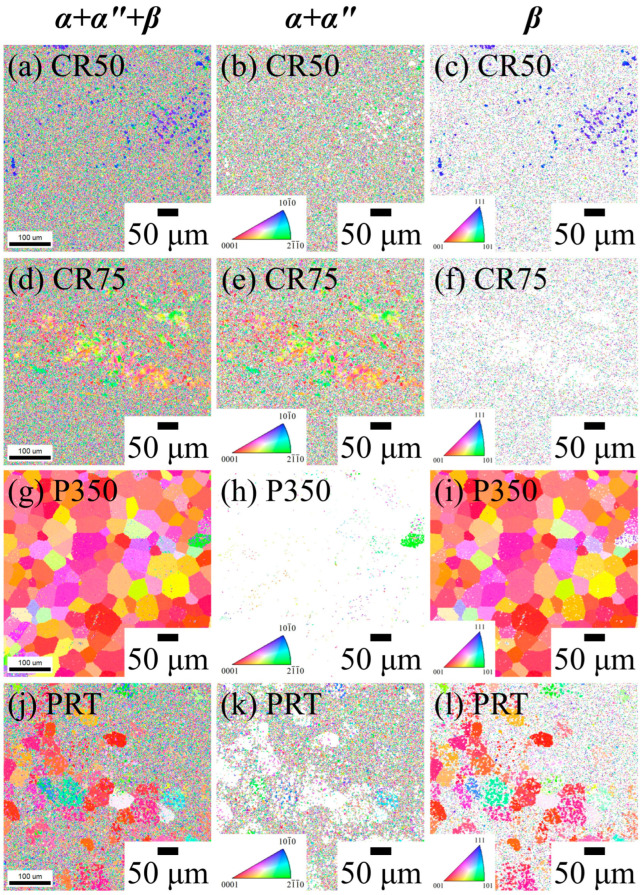
EBSD images (IPF and phase map) of Ti_80_–Nb_10_–Mo_5_–Sn_5_ at various conditions with multiphase.

EBSD images of the Ti_80_–Nb_10_–Mo_5_–Sn_5_ samples with a single β phase under various conditions are shown in Figure 2. All β-phase samples (ST, P150, P550, and P700) exhibited equiaxed grains, which is characteristic of the β phase and confirms the XRD results. The average grain size of the P150 (35.5 ± 5.3 μm) was similar to that of the ST (34 ± 5.0 μm) due to the lower heating temperature of the P150. In contrast, P550 and P700 under high-temperature treatment exhibited larger grain sizes (49.5 ± 9.9 and 54.5 ± 2.5 μm, respectively). The EBSD images of the Ti_80_–Nb_10_–Mo_5_–Sn_5_ samples with a non-single β phase in this study are shown in Figure 3. The grain size of the Ti_80_–Nb_10_–Mo_5_–Sn_5_ decreased significantly due to fragmentation after cold rolling. The average grain sizes of the β phase were <10 and <1 μm for CR50 and CR75, respectively. Furthermore, the CR75 had a larger grain size for the α″ phase (~5 μm) than the CR50 due to the induction of a greater α″ phase due to cold rolling. In P350 (Figure 3h), a small amount of the α phase precipitated, with the grain sizes of the β and α phases measuring 38.4 ± 6.1 and <1 μm, respectively. Additionally, a significant amount of the α phase was observed in the PRT sample (Figure 3k), confirming the precipitation of the α phase in the Ti_80_–Nb_10_–Mo_5_–Sn_5_ after ST and 30 days of precipitation hardening treatment at room temperature. Moreover, P350 and PRT had ultrafine grain sizes for the α phase (~1 μm), attributed to the short heating time (15 min) and low heating temperature (room temperature), respectively. This phenomenon can be attributed to the sluggish diffusion effect in high-entropy alloys, which impedes atomic diffusion and contributes to the formation of ultrafine α grains in the alloy [37].

### 3.2. Mechanical Properties

**Figure 4:**
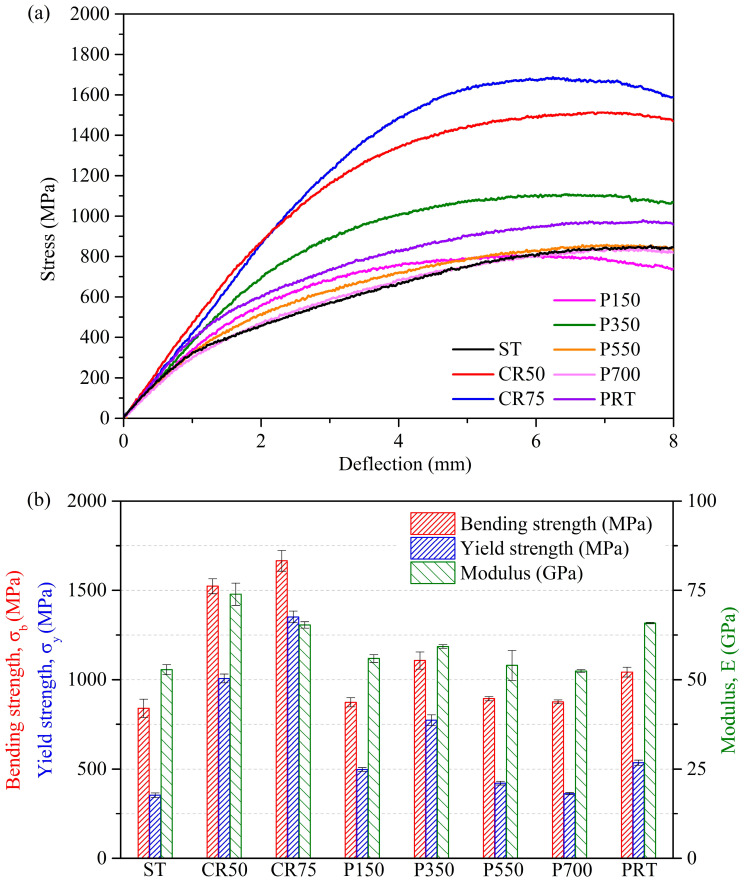
Mechanical properties of Ti_80_–Nb_10_–Mo_5_–Sn_5_ obtained using three-point bending tests. (**a**) Stress–deflection curves, and (**b**) bending strengths, yield strengths, and moduli.

The stress–deflection curves of the Ti_80_–Nb_10_–Mo_5_–Sn_5_ samples in various states under three-point bending tests are shown in Figure 4a. The comparison of the bending strengths, yield strengths, and elastic moduli of the Ti_80_–Nb_10_–Mo_5_–Sn_5_ samples after various treatments is illustrated in Figure 4b. Due to the contribution of work hardening and grain refinement, both CR50 and CR75 exhibited ultra-high yield strengths (1007 and 1351 MPa, respectively), which were far greater than ST (354 MPa), by 184% and 282%, respectively. Despite this, CR50 and CR75 still maintained a high bending deformation capability (deflection > 8 mm), a combination of the high strength and ductility often found in HEAs [38]. The high yield strengths of CR50 and CR75 are attributed to the work hardening induced by dislocation activity [12]. Additionally, cold rolling suppresses the dynamic recovery in the alloy, increasing the dislocation density as the grain size decreases, thereby enhancing the yield strength [12]. Among all heating conditions, P350 exhibited the highest yield strength (772 MPa) due to the strengthening of the ultrafine α precipitates. The yield strength of the Ti_80_–Nb_10_–Mo_5_–Sn_5_ after natural precipitation hardening treatment (536 MPa) was 51% higher than that of ST (354 MPa). However, the improvement in yield strength after natural precipitation hardening treatment is not as significant as that after cold rolling and certain annealing conditions. The experimental results demonstrate the possibility of enhancing mechanical properties using natural precipitation hardening treatment.

The elastic moduli of the Ti_80_–Nb_10_–Mo_5_–Sn_5_ increased by 40% and 24% after cold rolling of 50% (CR50) and 75% (CR75), respectively, due to the SIM transformation. It is well documented that the elastic modulus of the metastable β phase is lower than that of the α″ phase in Ti-Nb-based alloys [23,39,40]. Furthermore, the elastic anisotropy caused by cold rolling could have led to the increase in the elastic modulus of CR50 and CR75 [41]. The lower elastic modulus of CR75 compared to CR50 is attributed to an increase in the amount of cold rolling, which induced a greater α″ phase and increased dislocation density [42,43,44,45]. Overall, the bending strength and yield strength of the Ti_80_–Nb_10_–Mo_5_–Sn_5_ were improved significantly at the cost of a slightly increased elastic modulus after 75% cold rolling. Additionally, the elastic modulus of the Ti_80_–Nb_10_–Mo_5_–Sn_5_ slightly increased after the precipitation hardening treatment at 150 °C, which could be related to the increase in crystal defects caused by low-temperature heat treatment. When the heating temperature was further raised to 350 °C, the elastic modulus of the Ti_80_–Nb_10_–Mo_5_–Sn_5_ significantly increased to 59 GPa due to the fine precipitation of the α phase. Although the EBSD results (Figure 3) show that the size of the α phase in P350 was very fine, the α-phase precipitation did increase its elastic modulus, indicating that the α phase may not obtain a nanoscale size. When the heating temperature was further increased to 550° C and 700 °C, both the elastic moduli and yield strengths of the alloys decreased. Since P550 and P700 had a single β phase, the β-phase transus temperature of the Ti_80_–Nb_10_–Mo_5_–Sn_5_ may have been below 550 °C. Therefore, the annealing of the Ti_80_–Nb_10_–Mo_5_–Sn_5_ at 550 °C and 700 °C is similar to the effect of solution treatment, resulting in mechanical properties similar to those of the ST.

**Figure 5:**
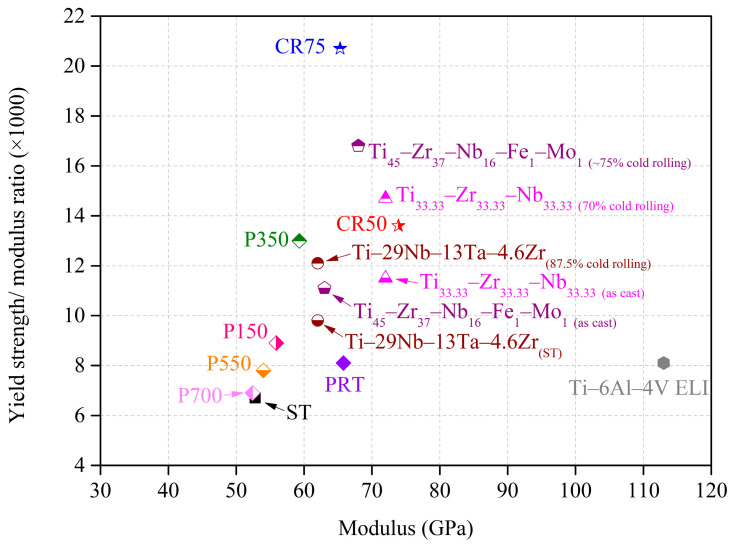
Yield strength/elastic modulus ratios (×1000) of Ti_80_–Nb_10_–Mo_5_–Sn_5_ at various conditions, a biomedical HEA [20], a biomedical MEA [47], and conventional Ti alloys [46].

The potential of an alloy as a biomedical implant can be evaluated by simultaneously comparing its mechanical strength and elastic modulus (E). An excellent biomedical implant requires a high yield strength (σ_y_) to prevent fracture within the body, coupled with a low elastic modulus (E) to avoid stress-shielding effects. Therefore, for the alloy’s elastic modulus (<80 GPa) to remain significantly lower than cortical bone, a higher σ_y_/E value indicates its greater potential as a biomedical implant. In Figure 5, the yield-strength-to-elastic-modulus ratio (σ_y_/E, ×1000) is presented for several materials, including conventional Ti alloys (Ti–6Al–4V ELI and Ti–29Nb–13Ta–4.6Zr, in wt%) [46], a biomedical HEA (Ti_45_–Zr_37_–Nb_16_–Fe_1_–Mo_1_, in at%) [20], a biomedical MEA (Ti_33.33_–Zr_33.33_–Nb_33.33_ in at%) [47], and the samples from this study. The CR75 had the highest σ_y_/E ratio (20.7), primarily attributed to its high σ_y_ value (1351 MPa), resulting from the combined effects of lattice distortion and grain refinement. Furthermore, P350 exhibited the highest σ_y_/E ratio (13) among all alloys with an elastic modulus below 60 GPa. The following section of this study focuses on the corrosion resistance of ST, CR75, and P350, elucidating their potential as biomedical implants.

### 3.3. Corrosion Properties

#### 3.3.1. Potentiodynamic Polarization Test

**Figure 6:**
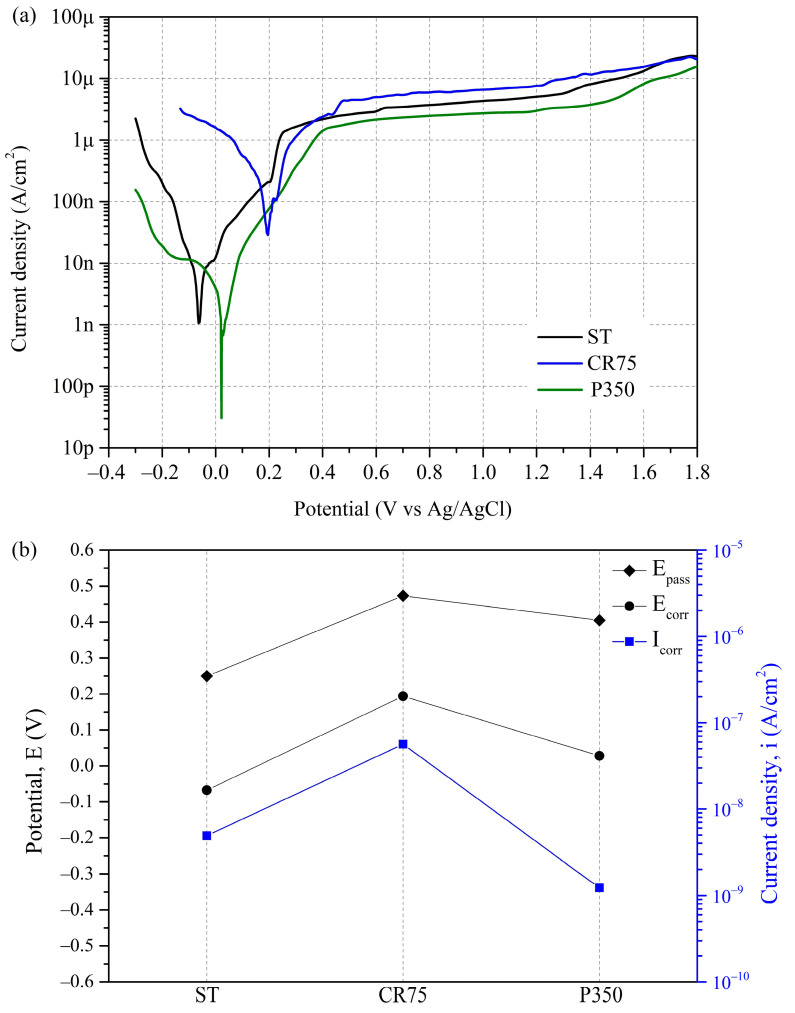
The polarization curves of Ti_80_–Nb_10_–Mo_5_–Sn_5_ at various conditions after potentiodynamic polarization tests in phosphate-buffered saline at 37 °C.

To understand the corrosion behavior of the Ti_80_–Nb_10_–Mo_5_–Sn_5_ in the human body after different treatments, potentiodynamic polarization tests were conducted on three kinds of alloys with different structures, including ST (β), CR75 (β + α″), and P350 (β + α). The polarization curves of ST, CR75, and P350 in PBS at 37 °C are shown in Figure 6a. In addition, the electrochemical parameters obtained from the potentiodynamic polarization tests, such as the corrosion potential (E_corr_), corrosion current density (i_corr_), passivation potential (E_pass_), passive current density (i_pass_), the slope of the anodic curve (β_a_), the slope of the cathode curve (β_c_), and polarization resistance (R_p_), are shown in Table 3. The trend charts of E_corr_, i_corr_, and E_pass_ for the three kinds of samples are shown in Figure 6b.

From the polarization curves of ST, CR75, and P350, it can be seen that the corrosion resistance of Ti_80_–Nb_10_–Mo_5_–Sn_5_ significantly increased after cold rolling or precipitation hardening treatment, and the pitting potentials of the three kinds of samples were all higher than 1.8 V. Among them, CR75 had the highest E_corr_ value (0.2 V), significantly higher than ST (–0.06 V) and P350 (0.02 V). Generally, alloys will generate a large number of defects on the surface and inside after cold rolling, and these high-energy defects will lead to a decrease in the corrosion resistance of the alloy [48]. In addition, the phase boundaries of multi-phase alloys are also high-energy areas that are prone to preferential corrosion. Furthermore, several studies have shown that the E_corr_ value of Ti alloys decreased significantly after cold working [48,49,50,51]. However, this study found that the E_corr_ value of the Ti_80_–Nb_10_–Mo_5_–Sn_5_ significantly increased after cold rolling. Some of the literature has noted that the increase in the E_corr_ value of the alloy after deformation is related to its ultrafine grain structure [17,52,53,54,55]. According to Martin et al. [54], the corrosion behavior of the alloy is concurrently influenced by both its microstructure and grain size. A microstructure with uniformly distributed fine grains exhibits superior corrosion resistance compared to needle-like or lamellar microstructures, attributed to the minimization of tendencies for microgalvanic cell formation. Hence, CR75 demonstrates the highest E_corr_ value, signifying its optimal corrosion resistance. On the other hand, the i_corr_ value of P350 (2.54 nA/cm^2^) was significantly lower than those of ST (9.02 nA/cm^2^) and CR75 (131.26 nA/cm^2^), indicating that P350 had the lowest corrosion rate. The low i_corr_ value of P350 is attributed to the precipitation of the α phase, which reduces the conductivity [56]. Additionally, P350 exhibited the highest R_P_ value (2409.44 kΩ cm^2^), attributed to its low i_corr_ value. Yet, the polarization resistances acquired using potentiodynamic polarization tests do not account for the effects of the electrolyte and capacitance. Further elucidation of the alloy’s corrosion resistance is necessary, using EIS testing.

The difference between the E_corr_ and E_pass_ values acts as an indicator for assessing an alloy’s passivation capability. Table 3 shows that the CR75 had the lowest E_corr_–E_pass_ value (0.27 V), indicating that the alloy can quickly undergo passivation after corrosion. The high passivation ability of CR75 is attributed to its ultrafine grain size. After grain refinement treatment, the increase in the grain boundary density and dislocation density can reduce the surface work function and promote the formation of thicker and less defective passivation films [17,55]. In addition, more grain boundaries can provide more nucleation sites for oxidation in the passivation layer [57,58].

#### 3.3.2. SEM Images

**Figure 7:**
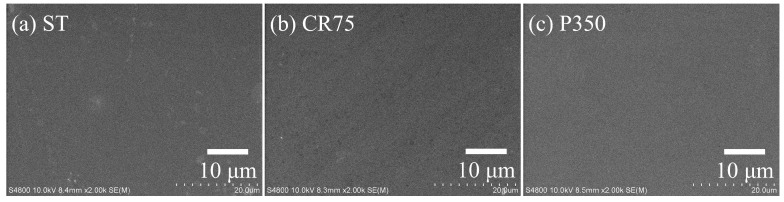
SEM photos of Ti_80_–Nb_10_–Mo_5_–Sn_5_ at various conditions after potentiodynamic polarization tests.

The SEM images of the ST, CR75, and P350 after potentiodynamic polarization testing are shown in Figure 7. The surfaces of all samples remained smooth after potentiodynamic polarization. No large corrosion products or pitting holes were observed. P350 (Figure 7c), with a high E_corr_ value and the lowest i_corr_ value, did not show any corrosion or products, attributed to the precipitation of the α phase. The ultrafine-grained α precipitates can fill the internal defects of grain boundaries and grains, which can inhibit intergranular corrosion and impede the permeation of electrolyte solutions, thereby enhancing the chemical stability of the alloy [28,29]. Small white corrosion products were observed on the surface of the ST (Figure 7a), not found in the other two samples, due to its low E_corr_ and high E_corr_–E_pass_ value. Several light corrosion stripes parallel to the rolling direction were observed on the surface of CR75 (Figure 7b), inferred to be the preferential corrosion of the residual stress regions generated by cold rolling. As a result, the CR75 had the highest i_corr_ value. Despite the limited number of stripes observed on the CR75 surface, these stripes could compromise the integrity of the passive film. Therefore, it is imperative to conduct further analysis of the chemical composition and characteristics of the passive film using EIS and XPS.

#### 3.3.3. EIS

**Figure 8:**
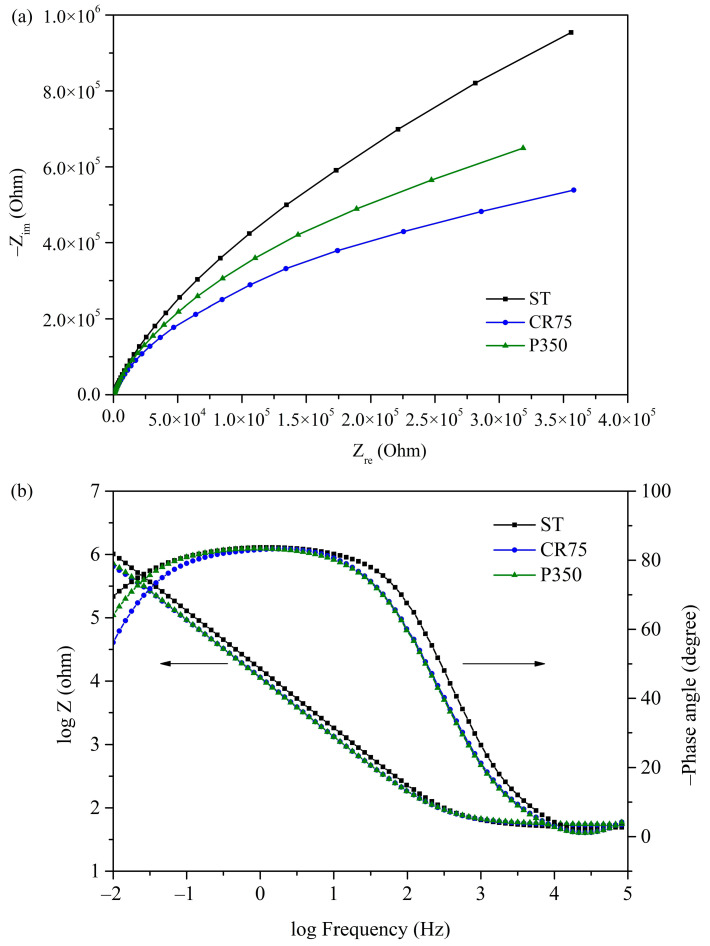
(**a**) Nyquist diagrams and (**b**) Bode plots of Ti_80_–Nb_10_–Mo_5_–Sn_5_ at various conditions in PBS solution at 37 °C.

Figure 8 presents the EIS results of ST, CR75, and P350 in PBS solution at 37 °C, with Nyquist diagrams and Bode plots at frequencies ranging from 10^5^ to 10^−2^ Hz shown in Figure 8a,b, respectively. From the Nyquist plot, it can be observed that the capacitance semicircles of all three samples exhibited an incomplete shape, which is related to the capacitive response of the passive film [59]. Compared to CR75 and P350, ST has a capacitance semicircle with a larger diameter, indicating that the passive film of the ST has the best corrosion resistance. From the Bode modulus curves, it can be seen that the slopes of the curves of all the samples in the high-frequency range (10^4^ to 10^5^ Hz) were close to 0, indicating that all samples have a similar electrolyte resistance [59]. Furthermore, all the Bode phase curves had a phase angle of approximately 0° at high frequencies, indicating no phase signal delay. In the low-frequency range (10^−1^ to 10^1^ Hz), the Bode phase slopes of all samples were close to 0, demonstrating the formation of a passive film on the surface of the samples. The negative phase angles of the Bode phase curves of all three samples were greater than 80°, indicating that the passive films generated in PBS exhibit a good capacitive behavior [60].

**Figure 9:**
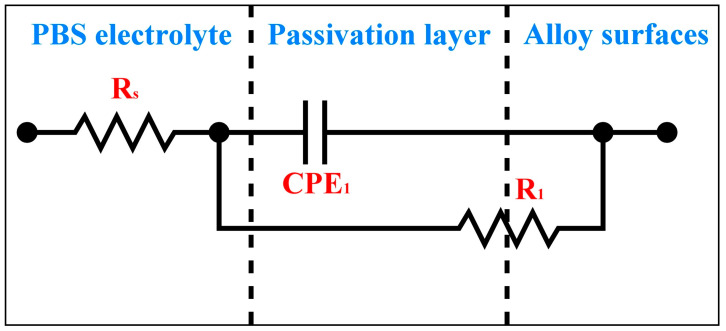
Equivalent electrical circuit (EEC) model of three Ti_80_–Nb_10_–Mo_5_–Sn_5_ samples.

An equivalent electrical circuit (EEC) model of the three Ti_80_–Nb_10_–Mo_5_–Sn_5_ samples is shown in Figure 9. The design of this EEC model is based on the results of the potentiodynamic polarization tests for ST, CR75, and P350, which did not experience pitting corrosion in the potential range of –0.3 to 1.8 V. Within this EEC model, R_s_ represents the electrolyte resistance, CPE_1_ denotes the constant phase element of the passive layer, and R_1_ stands for the polarization resistance of the passive layer. The EIS data of each sample, including the electrolyte resistance (R_s_), CPE_1_, R_1_, deviation parameter for CPE_1_ (n_1_), effective constant capacitance value (C_eff_), and chi-square value (χ^2^) fitted based on the EEC in Figure 9 using the EIS Spectrum Analyzer software, are listed in Table 4. The formula for calculating C_eff_ is as follows [59]: $C_{eff,1}={{CPE}_{1}}^{\frac{1}{n_{1}}}{R_{1}}^{\frac{1-n_{1}}{n_{1}}}$. The χ^2^ values of each sample were in the order of 10^−4^, indicating that the results of the equivalent circuit fitting are valid. The ST had the highest R_1_ value (3.96 × 10^6^ Ω cm^2^) and the lowest C_eff_ value (1.64 × 10^−5^ F cm^2^), indicating the best corrosion resistance of the passive film on the surface of ST. CR75 showed comparatively lower R_1_ and C_eff_ values owing to the compromised integrity of the passive layer resulting from the residual stress induced during cold rolling. The P350 exhibited a high E_corr_ value and the lowest i_corr_ value in the potentiodynamic test (Figure 6), but the R_1_ value of P350 in the EIS test was lower than that of ST. The precipitated phase (α) in P350 may hinder the diffusion of oxygen ions and slow down the growth rate of the passive layer [61,62]. Furthermore, the lower C_eff_ value of P350 compared to ST also indicates a thinner passive layer on the surface of P350. Nevertheless, ST, CR75, and P350 all exhibit ultra-high R_1_ values (>10^6^ Ω cm^2^) and low C_eff_ values (<3 × 10^−5^ F cm^2^), which are sufficient for the corrosion resistance requirements of biomedical implants.

#### 3.3.4. XPS

**Figure 10:**
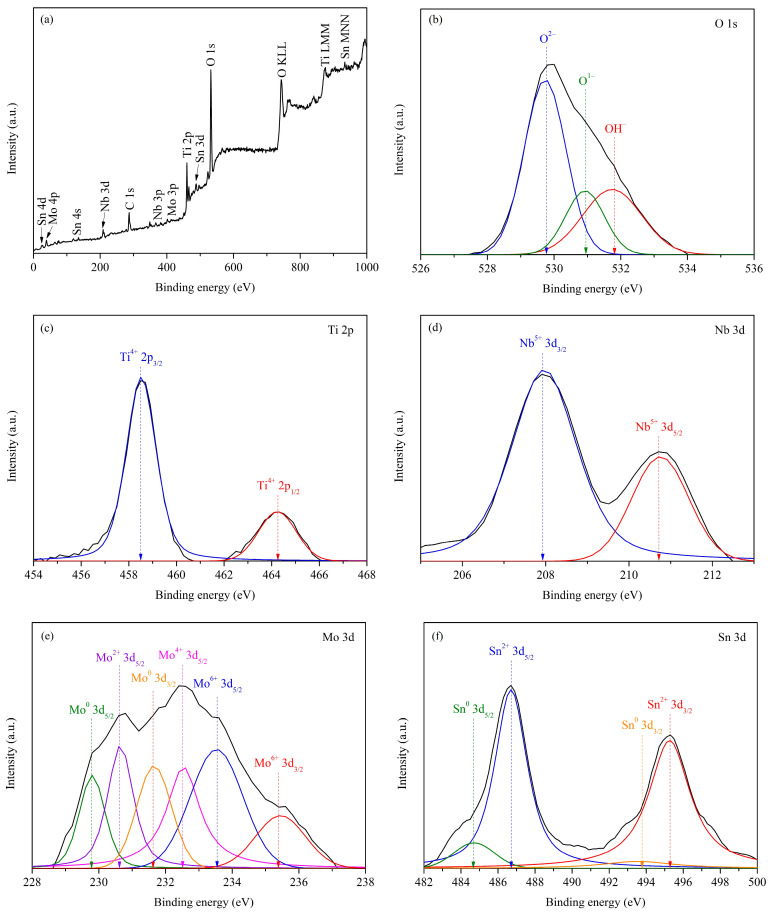
The chemical characterization of the surface of CR75 after potentiodynamic polarization test. (**a**) Full spectrum and (**b**–**f**) narrow scans.

The XPS chemical characterization results of the CR75 surface after potentiodynamic polarization are presented in Figure 10. The full spectrum (Figure 10a) indicates the presence of Ti, Nb, Sn, Mo, C, and O elements in the oxide film/passivation layer on CR75. The presence of C and O elemental peaks in the full spectrum result from surface carbon contamination and the oxide layer. Narrow scans of the O 1s, Ti 2p, Nb 3d, Sn 3d, and Mo 3d elements of the CR75 sample are shown in Figure 10b–f, respectively. The O 1s peaks correspond to the OH^−^, O^−^, and O^2−^ oxidation states; the Ti 2p peaks belong to the Ti^4+^ oxidation state; the Nb 3d peaks are related to the Nb^5+^ oxidation state; the Sn 3d spectrums are ascribed to the Sn^0^ and Sn^4+^ oxidation states; and the Mo 3d peaks are composed of the Mo^0^, Mo^2+^, Mo^4+^, and Mo^6+^ oxidation states. Therefore, the surface oxide film/passivation layer of the CR75 mainly comprises TiO_2_, Nb_2_O_5_, SnO_2_, MoO, MoO_2_, and MoO_3_. The presence of Nb_2_O_5_ improves the resistance to Cl^−^ ion erosion and enhances the structural integrity of the oxide film/passivation layer [48]. In the passivation layer of Ti alloys, there may be O vacancies and Ti defects, which will lead to a decrease in the corrosion resistance of the passivation layer. When ions with a larger radius, such as Nb^5+^ and Mo^6+^, are present in the alloy passivation layer, they can form a chemical bond with Ti atoms and eliminate Ti defects, thereby improving the corrosion resistance [63].

In contrast to the Ti, Nb, and Sn elements, Mo exhibits multiple oxidation states in the passivation layer of the CR75. The electron configuration and elemental properties of Mo affect the formation of its oxidation state. The electron configuration of Mo is [Kr]4d^5^5s^1^, in which 4d electrons have relatively high energy and easily participate in chemical reactions to form compounds, while the chemical properties of 5s electrons are relatively inert. Hence, in Ti-rich alloys with Mo as an alloying element, the electron configuration and elemental properties of Mo promote the formation of 2+, 3+, and 4+ oxidation states while impeding the formation of 6+ oxidation states. Moreover, other alloy elements present in the alloy (Ti, Nb, and Sn) compete with Mo elements and further limit the oxidation state of Mo. As a consequence, the existence of several oxidation states of Mo in the passivation layer of the CR75 is unrelated to its uneven surface. In summary, although the surface of the CR75 is not very uneven, the presence of Nb, Sn, and Mo oxides in the passivation layer still makes it have a high R_1_ value (>10^6^ Ω cm^2^) and low C_eff_ value (<3 × 10^−5^ F cm^2^).

## 4. Conclusions

The microstructure, mechanical properties, and corrosion properties of the Ti_80_–Nb_10_–Mo_5_–Sn_5_ alloy were investigated under different conditions, including cold rolling and precipitation hardening treatment at various temperatures. The XRD patterns revealed that the alloy exhibited a single β phase in the solution-treated state. In contrast, the SIM transformation induced a β + α″ phase after cold rolling (50% and 75%), while heating at 350 °C for 15 min resulted in a β + α phase. Cold rolling significantly reduced the grain size of the β phase, while high-temperature precipitation hardening treatment led to larger grain sizes. Additionally, α precipitates were found in the ST sample after precipitation hardening treatment at room temperature for 30 days. After undergoing 75% cold rolling, the Ti_80_–Nb_10_–Mo_5_–Sn_5_ alloy (CR75) exhibited a significant increase in yield strength, albeit with a slight increase in elastic modulus. Moreover, the corrosion resistance of the Ti_80_–Nb_10_–Mo_5_–Sn_5_ alloy significantly increased after both the cold rolling and precipitation hardening treatments. The CR75 exhibited the highest E_corr_ value, which was attributed to its ultrafine grain size and high passivation ability. The EIS results indicated ultra-high R_1_ values (>10^6^ Ω cm^2^) and low C_eff_ values (<3 × 10^−5^ F cm^2^) for the ST, CR75, and P350 samples, affirming their good corrosion resistance. The XPS analysis indicated that the surface oxide film/passivation layer of the CR75 mainly consisted of TiO_2_, Nb_2_O_5_, SnO_2_, MoO, MoO_2_, and MoO_3_. To summarize, CR75 displayed the highest σ_y_/E ratio alongside excellent corrosion resistance, positioning it as a promising candidate for biomedical implants.

## Figures and Tables

**Table 1:** Thermodynamic parameters of Ti-rich Ti_80_–Nb_10_–Mo_5_–Sn_5_ metastable medium-entropy alloy.

Alloy	ΔS_mix_ (J/K·mol)	ΔH_mix_ (KJ/mol)	δ	T_L_ (°C)	ρ (g/cm^3^)	[Mo]_eq_	VEC
Ti_80_–Nb_10_–Mo_5_–Sn_5_	5.89	–3.11	1.53	1740.8	5.34	14.44	4.2

**Table 2:** The codes of Ti_80_–Nb_10_–Mo_5_–Sn_5_ alloy under various conditions.

Code	Conditions
ST	Solution treatment (ST) (900 °C, 15 min)
CR50	ST + 50% cold rolling
CR75	ST + 75% cold rolling
P150	ST + precipitation hardening treatment (150 °C, 15 min)
P350	ST + precipitation hardening treatment (350 °C, 15 min)
P550	ST + precipitation hardening treatment (550 °C, 15 min)
P700	ST + precipitation hardening treatment (700 °C, 15 min)
PRT	ST + precipitation hardening treatment (25 °C, 30 day)

**Table 3:** Corrosion potential (E_corr_), corrosion current density (i_corr_), passivation potential (E_pass_), passive current density (i_pass_), the slope of the anodic curve (β_a_), the slope of the cathode curve (β_c_), and polarization resistance (R_p_) of Ti_80_–Nb_10_–Mo_5_–Sn_5_ under different conditions after potentiodynamic polarization tests.

	E_corr_ (V)	i_corr_ (nA/cm^2^)	E_pass_ (V)	i_pass_ (μA/cm^2^)	E_pass_–E_corr_ (V)	β_a_ (V)	β_c_ (V)	R_p_ (kΩ·cm^2^)
ST	–0.06	9.02	0.25	1.34	0.31	0.0132	0.0145	333.06
CR75	0.20	131.26	0.47	4.26	0.27	0.0072	0.0102	13.99
P350	0.02	2.54	0.41	1.47	0.39	0.0235	0.0351	2409.44

**Table 4:** The EIS data of Ti_80_–Nb_10_–Mo_5_–Sn_5_ under different conditions in the PBS electrolyte, including the electrolyte resistance (R_s_), constant phase element of the passive layer (CPE_1_), polarization resistance of the passive layer (R_1_), deviation parameter for CPE_1_ (n_1_), effective constant capacitance value (C_eff_), and chi-square value (χ^2^) fitted based on the EEC in Figure 9.

	R_s_ (Ω·cm^2^)	CPE_1_ (10^−5^ F·cm^2^)	R_1_ (10^6^ Ω·cm^2^)	n_1_	C_eff_ (10^−5^ F·cm^2^)	χ^2^ (10^−4^)
ST	51.615	1.1925	3.9566	0.923	1.6432	1.31
CR75	56.437	1.6504	1.4371	0.916	2.2072	2.32
P350	57.818	1.6527	2.2814	0.918	2.2856	2.28

## Data Availability

Data are contained within the article.
